# Patterns and timing of recurrence in esophageal squamous cell carcinoma patients treated with neoadjuvant chemoradiotherapy plus esophagectomy

**DOI:** 10.1186/s12885-021-08918-x

**Published:** 2021-11-09

**Authors:** Yushi Nagaki, Satoru Motoyama, Yusuke Sato, Akiyuki Wakita, Hiromu Fujita, Yoshihiro Sasaki, Kazuhiro Imai, Yoshihiro Minamiya

**Affiliations:** 1 Division of Esophageal Surgery, Akita University Hospital, Akita, Japan; 2 Department of Thoracic Surgery, Akita University Graduate School of Medicine, Akita, Japan; 3 Department of Comprehensive Cancer Control, Akita University Graduate School of Medicine, 1-1-1 Hondo, Akita, 010-8543 Japan

**Keywords:** TESCC, NACRT, TRG, Recurrence patterns, Recurrence timing

## Abstract

**Background:**

Tumor regression grade (TRG) after neoadjuvant therapy is reportedly predictive of prognosis in esophageal cancer patients, as lack of a response to neoadjuvant therapy is associated with a poor prognosis. However, there is little information available on the timing and pattern of recurrence after esophagectomy for thoracic esophageal squamous cell carcinoma (TESCC) that takes into consideration TRG after neoadjuvant chemoradiotherapy (NACRT). Here, in an effort to gain insight into a treatment strategy that improves the prognosis of NACRT non-responders, we evaluated the patterns and timing of recurrence in TESCC patients, taking into consideration TRG after NACRT.

**Methods:**

A total of 127 TESCC patients treated with NACRT and esophagectomy between 2009 and 2017 were enrolled in this observational cohort study. TRGs were assigned based on the proportion of residual tumor cells in the area (TRG1, ≥1/3 viable cancer cells; 2, < 1/3 viable cancer cells; 3, no viable cancer cells). We retrospectively investigated the timing and patterns of recurrence and the prognoses in TESCC patients, taking into consideration TRG after NACRT.

**Results:**

The 127 participating TESCC patients were categorized as TRG1 (42 patients, 33%), TRG2 (56 patients, 44%) or TRG3 (29 patients, 23%). The locoregional recurrence rate was higher in TRG1 (36.4%) patients than combined TRG2–3 (7.4%) patients. Patients with TRG3 had better prognoses, though a few TRG3 patients experienced distant recurrence. There were no significant differences in median time to first recurrence or OS among patients with locoregional or distant recurrence. There was a trend toward better OS in TRG2–3 patients with recurrence than TRG1 patients with recurrence, but the difference was not significant.

**Conclusions:**

NACRT non-responders (TRG1 patients) experienced higher locoregional recurrence rates and earlier recurrence with distant or locoregional metastasis. TRG appears to be useful for establishing a strategy for perioperative treatments to improve TESCC patient survival, especially among TRG1 patients. (303 words).

**Supplementary Information:**

The online version contains supplementary material available at 10.1186/s12885-021-08918-x.

## Background

Because most esophageal cancer patients are diagnosed with locally advanced tumors with lymph node metastasis, their prognosis is very poor. These patients are treated using multimodal therapy that includes surgery, radiotherapy and chemotherapy. Neoadjuvant chemotherapy (NAC) or neoadjuvant chemoradiotherapy (NACRT) are common strategies that have been shown to improve the prognosis of patients with advanced esophageal cancer [1–5]. Indeed, a recent meta-analysis found that NACRT followed by surgery has a survival benefit over surgery alone [6]. Moreover, several other meta-analyses suggest that, given its histopathological and/or long-term survival benefit, NACRT should be recommended over NAC to patients with esophageal cancer [7–9]. However, that suggestion remains controversial at present.

Histological evaluation of surgical specimens resected after neoadjuvant therapy can provide valuable information about the prognosis of patients with esophageal cancer. The earlier TNM staging system (7th edition) reflects the prognosis of patients who received upfront surgery [10], but the current TNM classification of the UICC (8th edition) added staging for patients who had received neoadjuvant therapy [11]. The ypStage reflects the prognosis of patients who received surgery after NAC or NACRT. The pathological tumor regression grade (TRG) after neoadjuvant therapy is the most commonly used and reliable system for assessing the response to therapy and patient prognosis [12, 13].

Currently, little is known about the pattern and timing of disease recurrence in patients with thoracic esophageal squamous cell carcinoma (TESCC) treated with NACRT followed by surgery. To improve the prognosis of these patients, it will be important to understand the association between the TRG achieved with NACRT and the patterns and timing of disease recurrence following surgery. In the present study, therefore, we evaluated the patterns and timing of recurrence in TESCC patients, taking into consideration TRG in an effort to gain insight into a treatment strategy that improves the prognosis of NACRT non-responders.

## Methods

### Patients

A total of 127 consecutive patients with confirmed TESCC treated with NACRT followed by curative surgery at Akita University Hospital between 2009 and 2017 were enrolled in this retrospective cohort study. Patients with a supraclavicular lymph node (cM1 lymph node) were included [14]. Clinical staging was done according to the TNM classification of the UICC (8th edition) [11], based on esophagogastroduodenoscopy (EGD), contrast-enhanced computed tomography (CE-CT), and positron emission tomography (PET-CT). Cervical and abdominal ultrasonography (US) and endoscopic ultrasound (EUS) were performed for staging, as necessary.

### Neoadjuvant chemoradiotherapy (NACRT)

NACRT was recommended to patients with either clinical T3–4 based on the depth of invasion by the primary tumor or regional lymph node metastasis (cT3–4 or cN+) and with an Eastern Cooperative Oncology Group performance status (ECOG PS) of 0. The NACRT protocol entailed radiotherapy (40.0–41.4 Gy in 20–23 fractions; 1.8–2.0 Gy/day, 5 days/week) with two courses of combined chemotherapy composed of 5-fluorouracil 800 mg/m^2^/day on days 1–5 and cisplatin (FP) or nedaplatin (FGP) 80 mg/m^2^/day on day 1. High-energy X-rays (10 MV) were used for the radiotherapy. All patients underwent 3-dimensional radiotherapy planning, and the radiotherapy target was set around the gross tumor volume and metastatic lymph nodes. As a result, in nearly all patients the upper-to-lower mediastinum was included in the irradiated fields.

### Surgery

Esophagectomy was scheduled to be performed more than 3 weeks after completing NACRT, by which time patients had no treatment-related adverse events worse than grade 2 according to the Common Terminology Criteria for Adverse Events (CTCAE) Version 4.0 [15]. Esophagectomy under right thoracotomy or thoracoscopic (including robot-assisted thoracoscopic) esophagectomy with 3-field lymph node dissection (bilateral cervical [including supraclavicular], mediastinal, and abdominal lymph nodes) was performed. In most patients, reconstruction was done with a gastric tube in open surgery via the posterior mediastinal or retrosternal route. In the remaining patients, laparoscopic (including robot-assisted laparoscopic) reconstruction using a pedicled colon was introduced.

### Pathological analysis

Surgically resected TESCC specimens were subjected to routine pathological examination. The level of tumor regression in response to preoperative therapy was evaluated based on the Japanese Classification of Esophageal Cancer [16, 17]. TRG was classified into four categories: TRG0, no recognized cytological or histological therapeutic effect; TRG1, slightly effective with apparently viable cancer cells accounting for 1/3 or more of the tumor tissue; TRG2, moderately effective with viable cancer cells accounting for less than 1/3 of tumor tissue; and TRG3, highly effective with no evidence of viable cancer cells. TRG defined according to the College of American Pathologists (CAP) cancer protocol is a commonly used and reliable system for assessing the response to therapy and patient prognosis [18]. In the present study, however, we used the classification defined by the Japanese Classification of Esophageal Cancer instead of the CAP cancer protocol to more objectively and clearly define the residual cancer (non-responder); e.g., TRG1 patients had more than 1/3 of their cancer remaining.

### Follow-up and treatment after recurrence

All patients visited our department every 2 months. At each visit, patients received a follow-up examination that included physical examinations and blood tests. Head, chest, and abdominal CE-CT was performed every 4 months during the first 3 years, then every 6 months during the next 2 years. EGD was performed yearly. These follow-up evaluations were performed for 5 years after the surgery or until the patient’s death. Depending on where recurrence first occurred, we classified it as locoregional or distant. Locoregional recurrences included regional lymph node metastasis within the surgical (lymph node dissected) field and intramural recurrence, whereas distant recurrences were defined as extra-regional lymph node metastasis, distant organ metastasis, or pleural or peritoneal dissemination. Depending on the individual case, recurrences were mainly treated with chemotherapy, though surgical resection and reradiation were also performed when possible.

### Statistical analysis

Survival time was defined as the duration between the surgery date and the event (death or recurrence) onset date. Continuous variables are presented as the median (range: minimum-maximum). Differences among the 3 TRG groups were analyzed using the chi-square test for non-continuous variables and the Kruskal-Wallis test for continuous variables. Overall survival (OS) was estimated using Kaplan-Meier curves, which were compared using the log-rank test. All statistical analyses were performed using JMP14 (SAS Institute, Cary, NC, USA). All *P* values were reported as two-sided with a significance level of 0.05.

## Results

### Pathological TRG and patient characteristics

The 127 participating TESCC patients were categorized as TRG0 (none, 0%), TRG1 (42 patients, 33%)), TRG2 (56 patients, 44%) or TRG3 (29 patients, 23%). Their characteristics were shown in Table 1. There were no differences among the three groups (excluding TRG 0) with respect to age, gender, tumor location, depth of invasion, lymph node metastasis, clinical stage, or tumor differentiation. TRG3 significantly correlated with downstaging (from cStage to ypStage) after NACRT (*P* < 0.0001).

**Table 1:** Patient characteristics at each tumor regression grade

	TRG1	TRG2	TRG3	***P***
**Number**				
	42 (33%)	56 (44%)	29 (23%)	
**Age at surgery**				0.6208
	65 (45–75)	63 (41–75)	64 (44–77)	
**Sex**				0.7033
**Female**	5 (12%)	10 (18%)	5 (17%)	
**Male**	37 (88%)	46 (82%)	24 (83%)	
**Tumor location**				0.9615
**Upper**	9 (21%)	12 (22%)	5 (17%)	
**Middle**	20 (48%)	27 (48%)	13 (45%)	
**Lower**	13 (31%)	17 (30%)	11 (38%)	
**Depth of invasion (cT)**				0.5727
**T1**	1 (2%)	2 (4%)	2 (7%)	
**T2**	1 (2%)	4 (7%)	3 (10%)	
**T3**	40 (95%)	48 (86%)	23 (79%)	
**T4**	0 (0%)	2 (4%)	1 (4%)	
**Lymph node metastasis (cN)**				0.9545
**N0**	4 (10%)	4 (7%)	3 (10%)	
**N1**	24 (57%)	33 (59%)	19 (66%)	
**N2**	13 (31%)	18 (32%)	7 (24%)	
**N3**	1 (2%)	1 (2%)	0 (0%)	
**Clinical stage (cStage)**				0.8519
**IIA**	4 (10%)	4 (7%)	3 (10%)	
**IIB**	2 (5%)	6 (11%)	5 (17%)	
**IIIA**	22 (52%)	26 (47%)	13 (45%)	
**IIIB**	13 (31%)	17 (30%)	7 (24%)	
**IIIC**	1 (2%)	3 (5%)	1 (4%)	
**Tumor differentiation**				0.1977
**Not poorly**	38 (90%)	49 (88%)	22 (76%)	
**Poorly**	4 (10%)	7 (12%)	7 (24%)	
**Tumor stage after NACRT**				< 0.0001*
**Downstaged**	16 (38%)	30 (54%)	28 (97%)	
**No change**	21 (50%)	19 (34%)	1 (3%)	
**Upstaged**	5 (12%)	7 (13%)	0 (0%)	
**Recurrence**				0.0113*
**Presence**	22 (52%)	22 (39%)	5 (17%)	
**Absence**	20 (48%)	34 (61%)	24 (83%)	
**Prognosis**				0.0316*
**Alive**	22 (52%)	37 (66%)	25 (86%)	
**Dead with ESCC**	18 (43%)	15 (27%)	3 (10%)	
**Dead with other diseases**	2 (5%)	4 (7%)	1 (4%)	

TRG, tumor regression grade; *, Considered significant

### Recurrence patterns

**Fig. 1:**
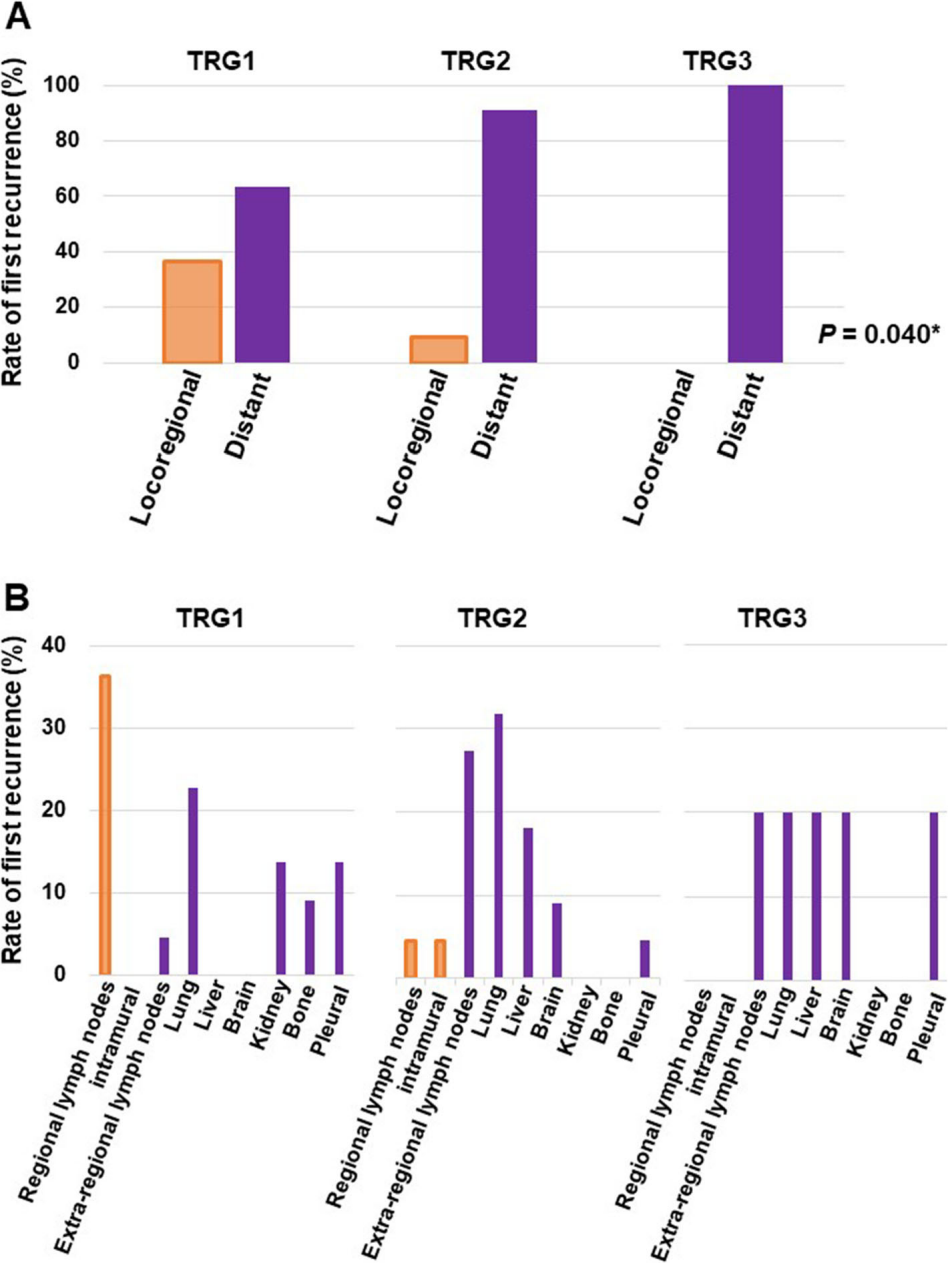
(A) Rates of first recurrence as locoregional (orange) or distant (purple) metastasis after neoadjuvant chemoradiotherapy (NACRT) followed by surgery for thoracic esophageal squamous cell carcinoma (TESCC). Bars depict the rate in each TRG group. (B) Rates of first recurrence at the indicated sites in each TRG group

Recurrence was detected in 38.6% (49 patients) of all patients, including 52% (22patients) of TRG1 patients (Table 1). Among them, 39 patients (79.6%) had distant recurrence, and 10 (20.4%) had locoregional recurrence (Table 2). The patterns of first recurrence segregated based on TRG are shown in Table 2 and Fig. 1A. Fourteen (63.6%) TRG1, 20 (90.9%) TRG2, and 5 (100%) TRG3 patients had distant recurrence, while 8 (36.4%) TRG1 and 2 (9.1%) TRG2 patients had locoregional recurrence. There were no TRG3 patients with locoregional recurrence, and the rate of locoregional recurrence was significantly (*P* = 0.040) higher in the TRG1 group than in the TRG2 or TRG3 group (Table 2).

**Table 2:** Recurrence patterns at each tumor regression grade

Patient	Locoregional	Distant	***P***
groups	recurrence	recurrence	
**All patients**	10 (20.4%)	39 (79.6%)	
**TRG1**	8 (36.4%)	14 (63.6%)	0.040*
**TRG2**	2 (9.1%)	20 (90.9%)	
**TRG3**	0 (0%)	5 (100.0%)	

TRG, tumor regression grade; *, Considered significant

Site recurrence rates in TRG1, 2, and 3 patients are shown in Fig. 1B. Distant metastasis occurred most frequently in the lung (33.3%) followed by an extra-regional lymph node (20.5%), liver (12.8%), pleural dissemination (12.8%), brain (7.7%), kidney (7.7%), and bone (5.1%). The most common site for distant recurrence in TRG1 and TRG2 patients was the lung (22.7 and 31.8%, respectively). Recurrence in an extra-regional lymph node was observed in all three groups but was detected most frequently in the TRG2 group, where it was detected in 27.3% of patients with recurrence. Sites of first recurrence in TRG3 patients were an extra-regional lymph node, lung, liver and brain as well as pleural dissemination.

### Recurrence timing

**Fig. 2:**
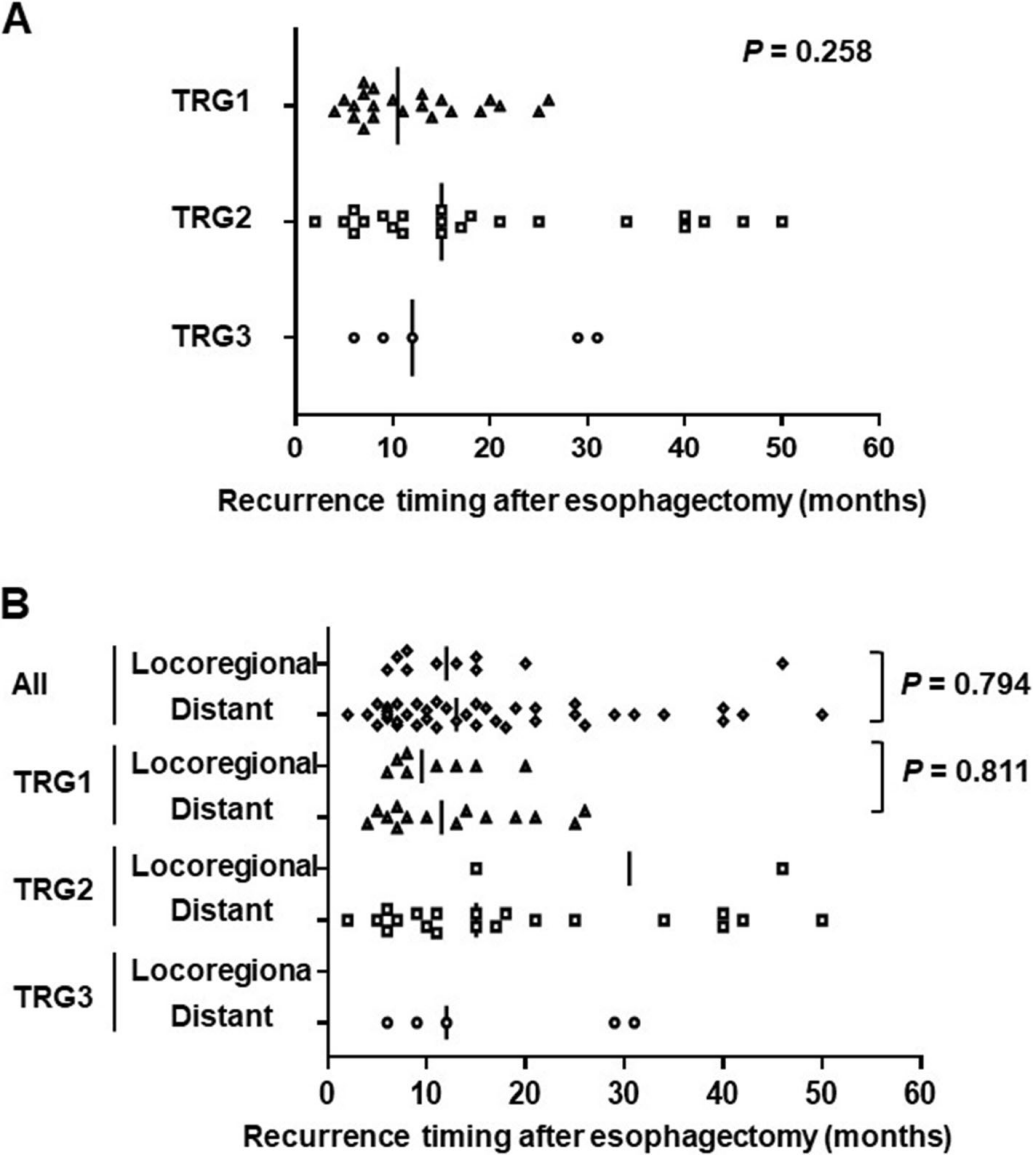
Plots indicating the times from surgery to recurrence in each TRG group without (A) and with (B) segregation of the patients based on whether their recurrence was locoregional or distant. In both panels, symbols represent individual patients with recurrence, and the vertical solid lines indicate the medians

The median time to the first recurrence was 10.5 (4–26), 15 (2–50) and 12 (6–31) months for TRG1, 2, and 3 patients, respectively (Table 3 and Fig. 2A). There was no significant difference in the median time to first recurrence among the three TRG groups (*P* = 0.258). The timing and frequency of locoregional and distant recurrence in each group are shown in Table 4 and Fig. 2B. There was no significant difference in median time to first locoregional or distant recurrence in all patients (*P* = 0.794) or in the TRG1 group (*P* = 0.811). Among the recurrent cases in each TRG group, about 50% of the patients experienced recurrence within the first year. The rates of recurrence within the first year were 55% (12/22 patients), 41% (9/22 patients), and 60% (3/5 patients) in the TRG1, TRG2, and TRG3 groups, respectively (*P* = 0.580).

**Table 3:** **Recurrence timing at each tumor regression grade**

Patient	Recurrence	Recurrance timing	***P***
groups	number		
**All patients**	49	13 (2–50)	
**TRG1**	22	10.5 (4–26)	0.258
**TRG2**	22	15 (2–50)	
**TRG3**	5	12 (6–31)	

**TRG, tumor regression grade**

**Recurrence timing is shown as the median (range) in months**

**Table 4:** Recurrence timing and frequency for each recurrence pattern at each tumor regression grade

Patient groups	Recurrence number		0–12 months	13–24 months	25–36 months	37–48 months	49–60 months	Median (months)
**TRG1**	All	22	12 (54.5%)	8 (36.4%)	2 (9.1%)	0	0	10.5 (4–26)
	Locoregional	8	5 (62.5%)	3 (37.5%)	0	0	0	9.5 (6–20)
	Distant	14	7 (50%)	5 (35.7%)	2 (14.3%)	0	0	11.5 (4–26)
**TRG2**	All	22	9 (40.9%)	6 (27.3%)	2 (9.1%)	4 (18.2%)	1 (4.5%)	15 (2–50)
	Locoregional	2	0	1 (50%)	0	1 (50%)	0	–
	Distant	20	9 (45%)	5 (25%)	2 (10%)	3 (15%)	1 (5%)	15 (2–50)
**TRG3**	All	5	3 (60%)	0	2 (40%)	0	0	12 (6–31)
	Locoregional	0	0	0	0	0	0	–
	Distant	5	3 (60%)	0	2 (40%)	0	0	12 (6–31)

TRG, tumor regression grade

### Prognosis

**Fig. 3:**
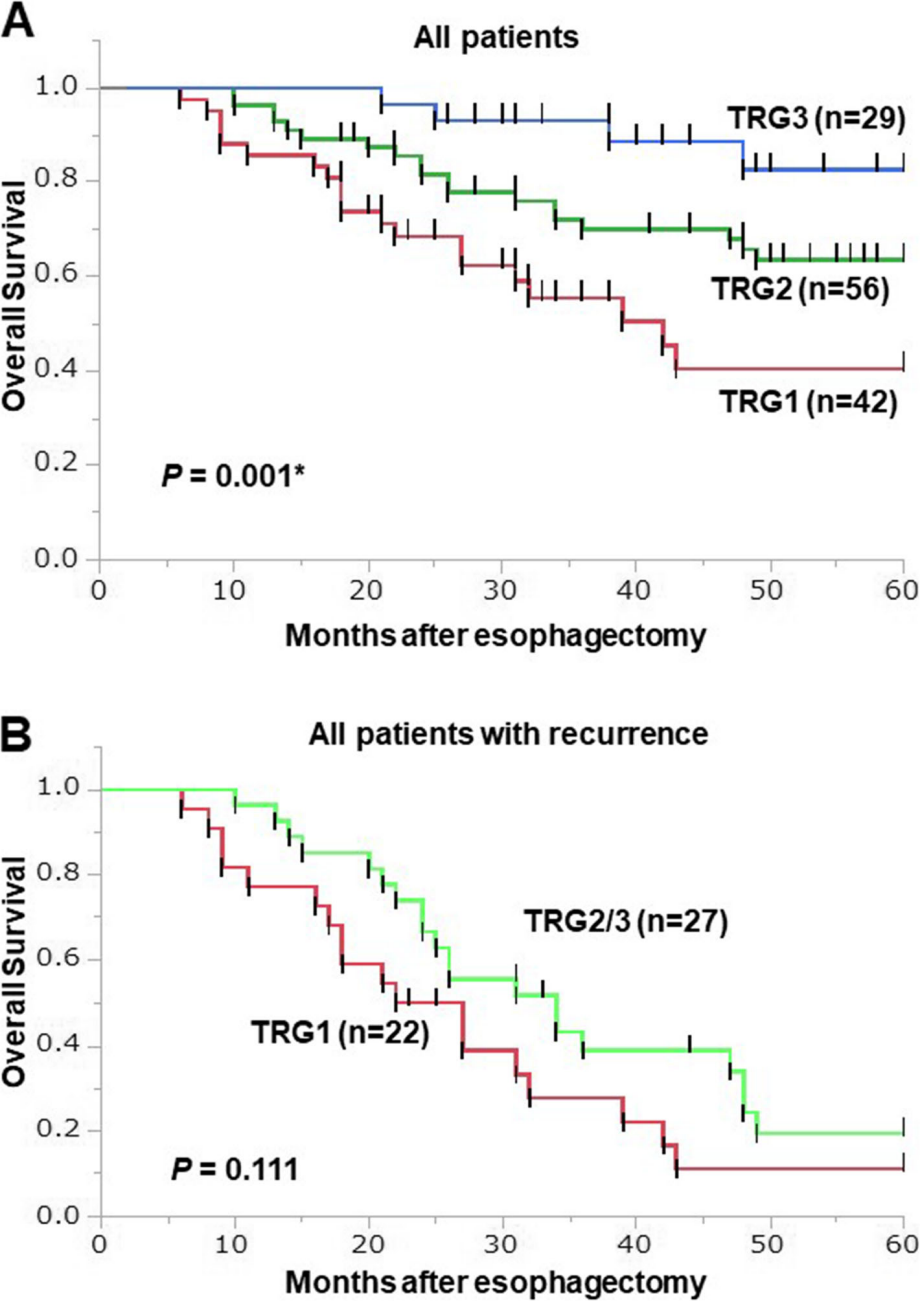
Kaplan-Meier survival curves showing OS in the TRG1 (Red, *n* = 42), TRG2 (Green, *n* = 56) and TRG3 (Blue, *n* = 29) groups (A). Kaplan-Meier survival curves comparing OS between TRG1 (red, *n* = 22) and TRG2/3 (green, *n* = 27) patients (B). The log-rank test was used to compare the two groups

**Fig. 4:**
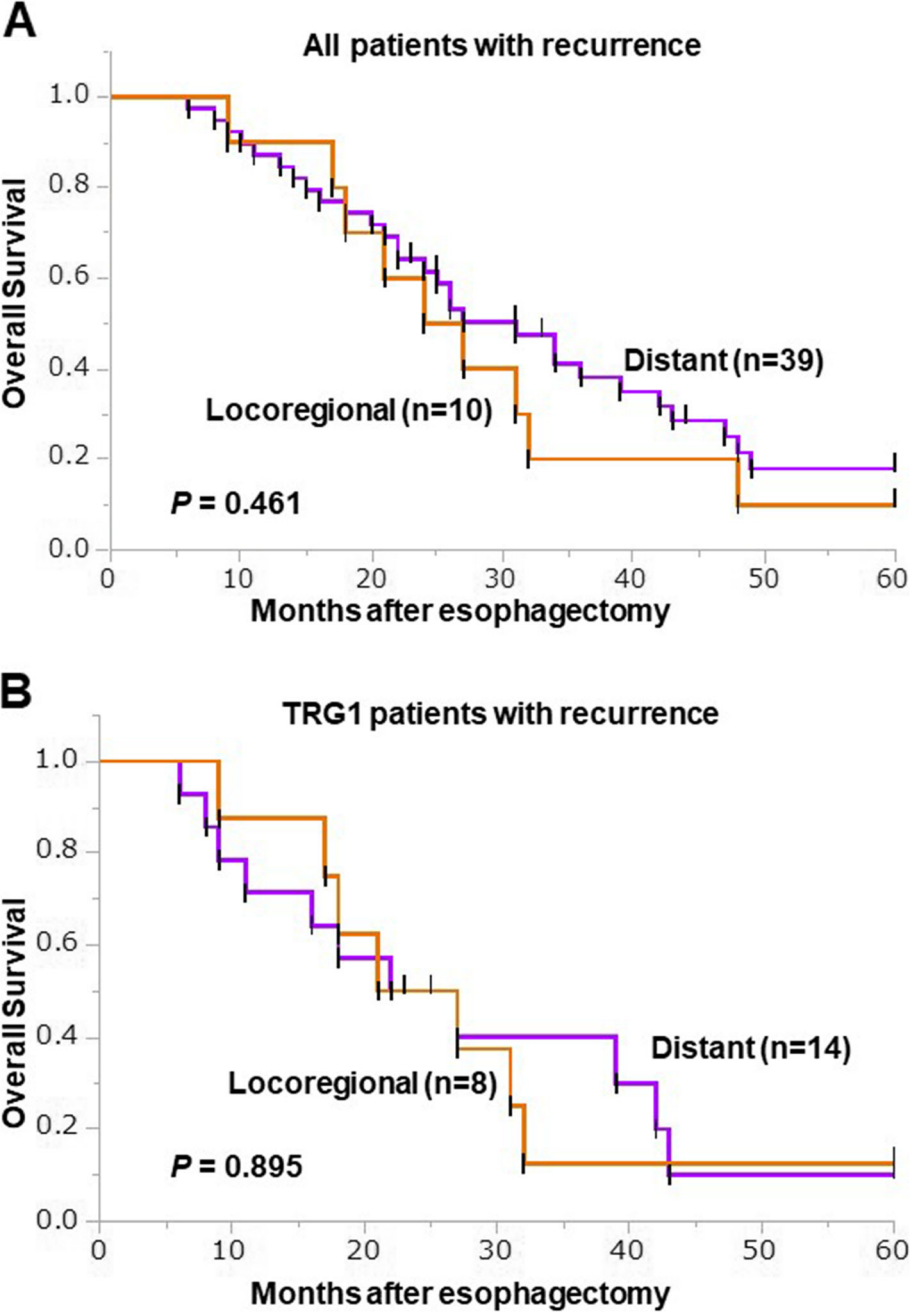
Kaplan-Meier survival curves comparing OS between patients with locoregional (orange, *n* = 10) or distant (purple, *n* = 14) recurrence: all patients (A), TRG1 patients (B). The log-rank test was used to compare the curves

There were significant differences with respect to recurrence and prognosis (*P* = 0.0113 and 0.0316, respectively) among the three TGR groups (Table 1). The median observation period after surgery for censored cases was 55.5 (18–132) months. Kaplan-Meier analysis showed that the 5-year OS rates among TRG1, TRG2 and TRG3 patients were 40.4, 63.6 and 82.8%, respectively (*P* = 0.001, Fig. 3A). Comparison between recurrent patients in the TRG1 group and the combined TRG2/3 group revealed a tendency toward better 5-year OS in recurrent patients in the TRG2/3 group than the TRG1 group, but the difference was not statistically significant (*P* = 0.111, Fig. 3B). Kaplan-Meier survival curves for OS among patients with distant and locoregional recurrence are shown in Fig. 4A and B. For all patients, there was no significant difference in 5-year OS between the locoregional and distant recurrence groups (*P* = 0.461, Fig. 4A). Similarly, within the TRG1 group, there was no significant difference in 5-year OS between patients with locoregional recurrence and those with distant recurrence (*P* = 0.895, Fig. 4B).

## Discussion

This study yielded several noteworthy results. First, distant metastasis was frequent in all TRG groups; however, the rate of locoregional recurrence was higher in the TRG1 group (36.4%) than the TRG2 or TRG3 group (9.1% or 0%). Second, there were no significant differences in median time to first recurrence among the three groups. The rates of recurrence within the first year were 55% (12/22 patients), 41% (9/22 patients), and 60% (3/5 patients) in the TRG1, TRG2, and TRG3 groups, respectively (*P* = 0.580). Third, there was a trend toward better OS in the combined TRG2/3 group than the TRG1 group. That said, patients with recurrence had poorer prognoses, irrespective of TRG or pattern of recurrence.

The development of multimodal therapies has improved the prognoses of esophageal cancer patients. A randomized controlled trial, the Chemoradiotherapy for Oesophageal Cancer Followed by Surgery Study (CROSS) trial, which compared outcomes after surgery plus NACRT with surgery alone in patients with esophageal cancer, found that NACRT plus surgery improved the survival rate among ESCC patients compared to esophageal adenocarcinoma (EAC) patients [19, 20]. In addition, the CROSS trial demonstrated that a pathological complete response (pCR) was seen in the resected specimens from 49% of ESCC patients in the NACRT plus surgery group [19]. Since that trial, many studies have shown that patients with a pCR after NACRT obtained a survival benefit, whereas non-pCR patients were associated with recurrence and poor prognoses after surgery [21, 22]. In the present study, TRG3 (in other words a pCR) was observed in 23% of ESCC patients. Differences in the backgrounds of the patients and the chemotherapy regimens used may explain the difference in the pCR rate between the CROSS trial and the present study. For instance, only 65% of the patients in the CROSS trial were positive for lymph node metastasis, whereas 91% of the patients were positive for lymph node metastasis prior to treatment in the present study. Nevertheless, the 5-year OS rate in this study was similar to or higher than in the CROSS trial. Thus, NACRT plus surgery, which improved local control of the esophageal tumor and regional metastatic lymph nodes and increased the rate of complete resection, is favored for esophageal cancer patients [7, 23].

Several studies have shown that NACRT followed by surgery reduces the locoregional recurrence rate compared to surgery alone [20, 24, 25]. Those studies reported that, after NACRT plus surgery, 12–22% of patients experienced locoregional recurrence, while 21–38% had locoregional recurrence after surgery alone. Moreover, the NEOCRTEC5010 trial, a multicenter phase III trial, reported that NACRT plus surgery was more effective than surgery alone for both locoregional control within the radiation field and distant recurrence [26]. On the other hand, Smit et al. [25] and the FFCD9901 trial [27] failed to detect a significant difference in distant metastasis-free survival between the NACRT plus surgery and surgery alone groups. Similarly, another randomized clinical trial comparing NACRT with NAC for treatment of cancer of the esophagus or gastroesophageal junction (ESCC and EAC) reported that there were no differences in the recurrence patterns between the treatment groups [28]. In the present study, we found that the first recurrence was locoregional in 20.4% of patients and was distant in 79.6% of patients. This suggests that NACRT followed by surgery enables control of local recurrence within the radiation and surgical fields, but whether NACRT affects control of distant recurrence remains unclear.

In this study, we focused on the patten and timing of recurrence after esophagectomy. Hagen et al. reported no significant difference in the time to locoregional or distant recurrence between complete and incomplete responders [29]. Oppedijk et al. reported that the majority of locoregional recurrences were within 2 years after surgery in ESCC or EAC patients treated with NACRT plus surgery, and there were no locoregional recurrences after 30 months [24]. In the present study, we found that about 50% of recurrences occurred within the first year, and most recurrences were within 3 years after surgery. However, because of the small number of recurrence cases, especially locoregional recurrences, in the TRG2 and TRG3 groups, it was difficult to compare the timing of locoregional and distant recurrences among the three TRG groups. The first recurrence tended to be earlier in the TRG1 group than the TRG2 group, irrespective of the recurrence pattern, but the difference was not significant.

Although the 5-year OS rates were better in the TRG2 and TRG3 groups than in the TRG1 group, 39% of TRG2 patients and 17% of TRG3 patients had recurrences. Like TRG1 patients, both TRG2 and TRG3 patients with recurrences had poor prognoses. The prognoses of TRG1 patients were poorer than those of TRG2 and TRG3 patients because TRG1 patients were more prone to recurrence. Recently, Kelly et al. reported a highly valued and important result from a global, randomized, double-blind, placebo-controlled phase III trial. They found that, compared to placebo, adjuvant nivolumab immunotherapy significantly improved median disease-free survival (22.4 months vs. 11.0 months, *p* < 0.001) after surgery in esophageal or gastro-esophageal junction cancer patients with residual pathological disease (ypT +/ypN+) after NACRT [30]. To improve the outcomes of TRG1 patients, it is essential to prevent recurrence after esophagectomy. Consequently, there is a need to establish new postoperative adjuvant treatment strategies, including use of an immune checkpoint inhibitor, like nivolumab.

Our study has several limitations. Its retrospective nature may have introduced selection bias. For this clinical study, 127 consecutive TESCC patients is a relatively small sample, but it is sufficient for an adequately powered statistical analysis. Moreover, although the impact is limited because there were few events (recurrences), our statistical analysis and conclusions take those circumstances into consideration. After recurrence, the treatments provided varied among the patients, and a small number of patients were provided immune checkpoint inhibitors, which we did not address in our analysis.

## Conclusions

TRG1 patients had early recurrence of both distant and locoregional metastasis and had poor prognoses. TRG2–3 patients had better prognoses, though the prognoses of patients with distant recurrences were poor. TRG status thus appears to be predictive of the pattern and timing of recurrence, which may be useful for establishing a strategy for perioperative treatment to improve TESCC patient survival.

## Supplementary Information

**Additional file 1.**

## Data Availability

The datasets used the current study are available from the corresponding author (YN) on reasonable request.

## Abbreviations

TRG: Tumor regression grade
TESCC: Thoracic esophageal squamous cell carcinoma
NACRT: Neoadjuvant chemoradiotherapy
NAC: Neoadjuvant chemotherapy
EGD: Esophagogastroduodenoscopy
CE-CT: Contrast-enhanced computed tomography
PET-CT: Positron emission tomography
US: Ultrasonography
EUS: Endoscopic ultrasound
ECOG PS: Eastern Cooperative Oncology Group performance status
CTCAE: The Common Terminology Criteria for Adverse Events
CAP: The College of American Pathologists
OS: Overall survival
CROSS trial: The Chemoradiotherapy for Oesophageal Cancer Followed by Surgery Study trial
EAC: Esophageal adenocarcinoma
pCR: pathological complete response

## References

[1] Kelsen DP, Winter KA, Gunderson LL, Mortimer J, Estes NC, Haller DG, Ajani JA, Kocha W, Minsky BD, Roth JA, Willett CG, Radiation Therapy Oncology Group, USA Intergroup (2007) Long-term results of RTOG trial 8911 (USA intergroup 113): a random assignment trial comparison of chemotherapy followed by surgery compared with surgery alone for esophageal cancer. J Clin Oncol 25(24):3719–3725, DOI: 10.1200/JCO.2006.10.4760. 10.1200/JCO.2006.10.4760 17704421

[2] Burmeister BH, Smithers BM, Gebski V, Fitzgerald L, Simes RJ, Devitt P, Ackland S, Gotley DC, Joseph D, Millar J, North J, Walpole ET, Denham JW (2005). Surgery alone versus chemoradiotherapy followed by surgery for resectable cancer of the oesophagus: a randomised controlled phase III trial. Lancet Oncol..

[3] Tepper J, Krasna MJ, Niedzwiecki D, Hollis D, Reed CE, Goldberg R, Kiel K, Willett C, Sugarbaker D, Mayer R (2008). Phase III trial of trimodality therapy with cisplatin, fluorouracil, radiotherapy, and surgery compared with surgery alone for esophageal cancer: CALGB 9781. J Clin Oncol.

[4] Ando N, Kato H, Igaki H, Shinoda M, Ozawa S, Shimizu H, Nakamura T, Yabusaki H, Aoyama N, Kurita A, Ikeda K, Kanda T, Tsujinaka T, Nakamura K, Fukuda H (2012). A randomized trial comparing postoperative adjuvant chemotherapy with cisplatin and 5-fluorouracil versus preoperative chemotherapy for localized advanced squamous cell carcinoma of the thoracic esophagus (JCOG9907). Ann Surg Oncol.

[5] Nakamura K, Kato K, Igaki H, Ito Y, Mizusawa J, Ando N, Udagawa H, Tsubosa Y, Daiko H, Hironaka S, Fukuda H, Kitagawa Y, Japan Esophageal Oncology Group/Japan Clinical Oncology Group (2013). Japan esophageal oncology group/Japan clinical oncology group: three-arm phase III trial comparing cisplatin plus 5-FU (CF) versus docetaxel, cisplatin plus 5-FU (DCF) versus radiotherapy with CF (CF-RT) as preoperative therapy for locally advanced esophageal cancer (JCOG1109, NExT study). Jpn J Clin Oncol.

[6] Kumar T, Pai E, Singh R, Francis NJ, Pandey M. Neoadjuvant strategies in resectable carcinoma esophagus: a meta-analysis of randomized trials. World J. Surg. Oncol. 18(1)59. 2020;18(1):59. 10.1186/s12957-020-01830-x. 10.1186/s12957-020-01830-x PMC7085863 32199464

[7] Zhao X, Ren Y, Hu Y, Cui N, Wang X, Cui Y (2018). Neoadjuvant chemotherapy versus neoadjuvant chemoradiotherapy for cancer of the esophagus or the gastroesophageal junction: a meta-analysis based on clinical trials. PLoS One.

[8] Kamarajah SK, Phillips AW, Ferri L, Hofstetter WL, Markar SR (2021). Neoadjuvant chemoradiotherapy or chemotherapy alone for oesophageal cancer: population-based cohort study. Br J Surg.

[9] Wang H, Tang H, Fang Y, Tan L, Yin J, Shen Y, Zeng Z, Zhu J, Hou Y, du M, Jiao J, Jiang H, Gong L, Li Z, Liu J, Xie D, Li W, Lian C, Zhao Q, Chen C, Zheng B, Liao Y, Li K, Li H, Wu H, Dai L, Chen KN (2021). Morbidity and mortality of patients who underwent minimally invasive esophagectomy after neoadjuvant chemoradiotherapy vs neoadjuvant chemotherapy for locally advanced esophageal squamous cell carcinoma: a randomized clinical trial. JAMA Surg.

[10] Sobin LH, Gospodarowicz MK, Wittekind C (2009). UICC International Union against Cancer: TNM classification of malignant Tumours, Seventh edition.

[11] Rice TW, Patil DT, Blackstone EH (2017). 8th edition AJCC/UICC staging of cancers of the esophagus and esophagogastric junction: application to clinical practice. Ann Cardiothorac Surg.

[12] Noble F, Nolan L, Bateman AC, Byrne JP, Kelly JJ, Bailey IS, Sharland DM, Rees CN, Iveson TJ, Underwood TJ, Bateman AR (2013). Refining pathological evaluation of neoadjuvant therapy for adenocarcinoma of the esophagus. World J Gastroenterol.

[13] Hatogai K, Fujii S, Kojima T, Daiko H, Kadota T, Fujita T, Yoshino T, Doi T, Takiguchi Y, Ohtsu A (2016). Prognostic significance of tumor regression grade for patients with esophageal squamous cell carcinoma after neoadjuvant chemotherapy followed by surgery. J Surg Oncol.

[14] Sato Y, Motoyama S, Wada Y, Wakita A, Kawakita Y, Nagaki Y, Terata K, Imai K, Anbai A, Hashimoto M, Minamiya Y (2021). Neoadjuvant Chemoradiotherapy followed by Esophagectomy with three-field lymph node dissection for thoracic esophageal squamous cell carcinoma patients with clinical stage III and with supraclavicular lymph node metastasis. Cancers..

[15] US Department of Health and Human Services (2009) Common Terminology Criteria for Adverse Events (CTCAE). Version 4.0. National Institutes of Health.

[16] Japan Esophageal Society (2017). Japanese classification of esophageal Cancer, 11th edition: part I. Esophagus.

[17] Japan Esophageal Society (2017). Japanese classification of esophageal Cancer, 11th edition: part II and III. Esophagus.

[18] Ryan R, Gibbons D, Hyland JM (2005). Pathological response following long-course neoadjuvant chemoradiotherapy for locally advanced rectal cancer. Histopathology..

[19] van Hagen P, Hulshof MC, van Lanschot JJ (2012). Preoperative chemoradiotherapy for esophageal or junctional cancer. N Engl J Med.

[20] Shapiro J, van Lanschot JJB, Hulshof MCCM, van Hagen P, van Berge Henegouwen M, Wijnhoven BPL, van Laarhoven H, Nieuwenhuijzen GAP, Hospers GAP, Bonenkamp JJ, Cuesta MA, Blaisse RJB, Busch ORC, ten Kate F, Creemers GM, Punt CJA, Plukker JTM, Verheul HMW, Bilgen EJS, van Dekken H, van der Sangen M, Rozema T, Biermann K, Beukema JC, Piet AHM, van Rij C, Reinders JG, Tilanus HW, Steyerberg EW, van der Gaast A, CROSS study group (2015). Neoadjuvant chemoradiotherapy plus surgery versus surgery alone for esophageal or junctional cancer (CROSS): long-term results of a randomized controlled trial. Lancet Oncol.

[21] Okumura H, Uchikado Y, Matsumoto M, Owaki T, Kita Y, Omoto I, Sasaki K, Sakurai T, Setoyama T, Nabeki B, Matsushita D, Ishigami S, Hiraki Y, Nakajo M, Natsugoe S (2013). Prognostic factors in esophageal squamous cell carcinoma patients treated with neoadjuvant chemoradiation therapy. Int J Clin Oncol.

[22] Schneider PM, Baldus SE, Metzger R, Kocher M, Bongartz R, Bollschweiler E, Schaefer H, Thiele J, Dienes HP, Mueller RP, Hoelscher AH (2005). Histomorphologic tumor regression and lymph node metastases determine prognosis following neoadjuvant radiochemotherapy for esophageal cancer: implications for response classification. Ann Surg.

[23] Courrech Staal EF, Aleman BM, Boot H (2010). Systematic review of the benefits and risks of neoadjuvant chemoradiation for oesophageal cancer. Br J Surg.

[24] Oppedijk V, van der Gaast A, van Lanschot JJ (2014). Patterns of recurrence after surgery alone versus preoperative chemoradiotherapy and surgery in the CROSS trials. J Clin Oncol.

[25] Smit JK, Güler S, Beukema JC, Mul VE, Burgerhof JGM, Hospers GAP, Plukker JTM (2013). Different recurrence pattern after neoadjuvant chemoradiotherapy compared to surgery alone in esophageal cancer patients. Ann Surg Oncol.

[26] Liu S, Wen J, Yang H, Li Q, Chen Y, Zhu C, Fang W, Yu Z, Mao W, Xiang J, Han Y, Zhao L, Liu H, Hu Y, Liu M, Fu J, Xi M (2020). Recurrence patterns after neoadjuvant chemoradiotherapy compared with surgery alone in oesophageal squamous cell carcinoma: results from the multicenter phase III trial NEOCRTEC5010. Eur J Cancer.

[27] Robb WB, Messager M, Dahan L, Mornex F, Maillard E, D'Journo XB, Triboulet JP, Bedenne L, Seitz JF, Mariette C, Fédération Francophone de Cancérologie Digestive, Société Française de Radiothérapie Oncologique, Union des Centres de Lutte Contre le Cancer, Groupe Coopérateur Multidisciplinaire en Oncologie., French EsoGAstric Tumour working group - Fédération de Recherche En Chirurgie. (2016) Patterns of recurrence in early-stage oesophageal cancer after chemoradiotherapy and surgery compared with surgery alone. Br J Surg 103(1):117–125, DOI: 10.1002/bjs.9959. 10.1002/bjs.9959 26511668

[28] von Döbeln GA, Klevebro F, Jacobsen AB, Johannessen HO, Nielsen NH, Johnsen G, Hatlevoll I, Glenjen NI, Friesland S, Lundell L, Yu J, Nilsson M (2019). Neoadjuvant chemotherapy versus neoadjuvant chemoradiotherapy for cancer of the esophagus or gastroesophageal junction: long-term results of a randomized clinical trial. Dis Esophagus.

[29] van Hagen P, Wijnhoven BP, Nafteux P (2013). Recurrence pattern in patients with a pathologically complete response after neoadjuvant chemoradiotherapy and surgery for oesophageal cancer. Br J Surg.

[30] Kelly RJ, Ajani JA, Kuzdzal J, Zander T, van Cutsem E, Piessen G, Mendez G, Feliciano J, Motoyama S, Lièvre A, Uronis H, Elimova E, Grootscholten C, Geboes K, Zafar S, Snow S, Ko AH, Feeney K, Schenker M, Kocon P, Zhang J, Zhu L, Lei M, Singh P, Kondo K, Cleary JM, Moehler M (2021). Adjuvant Nivolumab in resected esophageal or gastroesophageal junction Cancer. N Engl J Med.

